# Long-term follow-up of surgical treatment of thyroid-associated orbitopathy restrictive strabismus

**DOI:** 10.3389/fendo.2022.1030422

**Published:** 2022-11-10

**Authors:** Gustavo Savino, Roberta Mattei, Annabella Salerni, Claudia Fossataro, Pia Clara Pafundi

**Affiliations:** ^1^ Department of Ophthalmology, Fondazione Policlinico Universitario A. Gemelli IRCCS, Rome, Italy; ^2^ Facility of Epidemiology and Biostatistics, Gemelli Generator, Fondazione Policlinico Universitario A. Gemelli Istituti di Ricovero e Cura a Carattere Scientifico (IRCCS), Rome, Italy

**Keywords:** thyroid associated orbitopathy, muscle recession, diplopia, strabismus in TAO surgery, restrictive strabismus treatment

## Abstract

**Objective:**

Thyroid-associated orbitopathy (TAO) is the most frequent cause of extraocular muscle enlargement, with consecutive restrictive strabismus. The main muscles involved are inferior and medial rectus, resulting in horizontal esotropia and/or vertical strabismus. Surgery may either establish or improve binocular single vision. The aim of the present study is to describe long-term follow-up of patients who underwent horizontal or vertical TAO strabismus surgery.

**Methods:**

This observational retrospective study included 29 patients suffering from either vertical or horizontal TAO strabismus and diplopia, of whom 11 underwent bilateral medial recti muscle recession (Group A) and 18 underwent unilateral inferior rectus muscle recession (Group B). The endpoint of the study was the assessment of changes in deviation angle and diplopia across four time points (baseline, 7 days, 6 months, and 24 months) in each group.

**Results:**

In Group A, the horizontal deviation angle significantly decreased 7 days after intervention (*p* < 0.001), without modifications overtime. In Group B, both deviation angles in primary and down-gaze position significantly decreased from baseline, both 7 days after surgery (*p* < 0.001) and at 6 months (*p* = 0.040). An overcorrection, with an inversion of vertical deviation angle, was observed across the different time points.

**Conclusions:**

Horizontal TAO strabismus correction leads to significant improvements of deviation angle and diplopia, with a stable undercorrection overtime. Inferior rectus recession leads to more unstable results, with a trend towards overcorrection limited to the first 6 months after surgery.

## Introduction

Thyroid-associated orbitopathy (TAO) is the most frequent cause of either single or multiple extraocular muscle (EOM) enlargement at the core of restrictive strabismus (1).

Active TAO is characterized by inflammation and infiltration of orbital tissues by immune cells (T lymphocytes, mast cells, and B lymphocytes) and orbital remodeling, which gradually stabilizes and leads to the inactive phase of the disease (2).

EOMs are the major site of the disease process, and the impairment of ocular motility is caused by inflammation, followed by relatively rapid fibrosis of the involved muscles, with subsequent reduced elasticity and usually preserved muscle contractility (3).

The inferior rectus and medial rectus muscles are the most commonly involved, resulting in horizontal esotropia and/or vertical strabismus.

Surgery mainly aims either to establish or to improve the binocular single vision field (BSVF) in primary gaze and reading position. In addition, the evaluation of fusional vergences would be useful to predict the chance of postoperative compensation of possible hypo- or hypercorrection (4).

Unfortunately, achieving an optimal outcome may be challenging, especially in patients with combined horizontal and vertical deviations (5–8). The success rate of strabismus surgery in TAO patients is extremely variable and reported reoperation rate is approximately 45% of cases (7, 9, 10).

Undercorrection is the most common reported complication of horizontal strabismus correction (11, 12), and late overcorrection may occur after inferior rectus recession (8, 13).

Only a few studies have assessed the long-term effects of surgical treatment of TAO associated with strabismus in subsets of patients with thyroid function within the reference ranges, although they separately assess horizontal and vertical treatment (9, 11). The aim of the present study is to describe the long-term follow-up of patients submitted to either horizontal or vertical TAO strabismus surgery. Postoperative motor and sensory outcomes, and margin reflex distance 2 (MRD2) in patients submitted to inferior rectus weakening were also assessed.

## Materials and methods

### Study design and population

This observational retrospective cohort study included all patients suffering from either vertical or horizontal TAO strabismus and diplopia and surgically treated at the Ophthalmology Unit of the Fondazione Policlinico Universitario A. Gemelli IRCCS, Rome (Italy), between 1 January 2011 and 31 January 2016.

Patients previously medically or surgically treated, decompressed, with a follow-up period of less than 48 months, as well as subjects with either missing or incomplete records were excluded.

All patients signed a written informed consent to use their data for research aims. Photographs were obtained in selected cases with patients’ permission. The study was carried out with approval from the Head and Neck Institutional Review Board (approval ID; 18/2020) and in accordance with the 1976 Declaration of Helsinki and its later amendments.

### Procedures

All patients underwent a complete orthoptic and ophthalmological assessment, including best-corrected visual acuity (BCVA) measurement, near (33 cm) and far (6 m) prism and alternating cover test (PACT), and ocular motility evaluation.

Divergence and convergence fusional amplitudes (FAs) were measured, when possible, at distance and at near fixation through the full optical correction. An accommodative target was used first at distance (6 m) and then at near (33 cm). Starting from the base-out or base-up or down prisms totally compensating the deviation, base-out prisms of decreasing power and of increasing power were used to measure divergence and convergence FA, respectively. All surgical procedures were performed in our clinic by two experienced surgeons (GS and AS).

Forced duction test (FDT) was performed intraoperatively, before surgery, under general anesthesia. The eye was moved with two-toothed forceps applied to the conjunctiva at the limbus towards the opposite direction to that in which mechanical restriction was suspected. To evaluate the presence of mechanical restriction involving the inferior rectus, forceps were applied at the 3- and 9-o’clock positions, and the eye was moved in sursumduction (i.e., upward rotation of an eye).

All patients underwent intraoperative relaxed muscle positioning technique. Muscles were recessed to the positions where they rested freely on the globe without tension; absorbable not adjustable sutures were placed. The conjunctiva was further recessed. Moreover, an infratarsal lower eyelid retractor lysis was performed at the same time as inferior rectus muscle recession to reduce the chance of lower eyelid retraction.

### Endpoints

The primary endpoint of the study was the assessment of deviation angle modifications at a 2-year follow-up, across four time points (baseline, 7 days, 6 months, and 24 months) in the two subgroups, i.e., patients who underwent bilateral medial rectus recession (Group A) and patients who underwent unilateral inferior rectus recession (Group B). As secondary endpoints, changes in postoperative motor and sensory outcomes (i.e., all types of diplopia) were assessed. Also, only in patients submitted to inferior rectus weakening (Group B) were MRD2 changes at 6 months also evaluated.

### Statistical analysis

All variables were first analyzed by descriptive statistic techniques. In-depth, qualitative variables were described as absolute and percentage frequencies. The Shapiro–Wilk test was applied to assess the distribution of quantitative variables. Data were then expressed either as mean and standard deviation (SD), whether normally distributed, or as median and interquartile range (IQR), otherwise.

Between groups, differences were assessed by the Fisher exact test or the Chi-squared test, with Yates correction, as appropriate, on qualitative variables. Quantitative data, indeed, were evaluated, either by the Student’s *t*-test or the non-parametric Mann–Whitney *U* test.

Changes in the deviation angle across the four time points (baseline, 7 days, 6 months, and 24 months) in each subgroup were instead analyzed either by an ANOVA for repeated measures or Friedman test. MRD2 modification at 6 months from pre-surgery in Group B was instead assessed by a paired Student’s *t*-test, due to the Gaussian distribution of the data. Finally, modifications in all forms of diplopia since the immediate post-surgery (7 days *vs*. 6 months and 7 days *vs*. 24 months) in each subgroup were instead assessed by the McNemar or the Cochran *Q* tests, as appropriate. Data of pre-surgery were not considered, as all of the patients had diplopia.

A *p*-value <0.05 was considered as statistically significant. All analyses were performed by using R software version 4.1.2 (CRAN ^®^, R Core 2021) and STATA version 16 (STATA Corp).

## Results

Twenty-nine patients were included in the study: 11 underwent bilateral medial recti muscle recession and 18 underwent unilateral inferior rectus muscle recession.

The two subgroups were homogeneously distributed as for age and sex, as well as for smoking habit and hormonal compensation. Thyroid hormone levels were within the normal range in 72.7% of patients of Group A and in 94.4% patients of Group B. All patients had thyroidectomy/radioactive iodine treatment, except from three in Group A, treated with anti-thyroid drugs.

As for diplopia, this was present in all patients in the constant form. Focusing on Group B, diplopia associated with vertical inferior deviation angle was constant in 14 of 18 cases (77.8%).

At 7 days since operation, divergence and convergence FA for near and for distance were measured, with insignificant difference between the two subgroups. All data are reported in **Table 1**.

**Table 1:** Baseline general characteristics of the study cohort (*n* = 29).

	Group A(*n* = 11)	Group B(*n* = 18)	*p*
Age (years), mean (SD)	56.9 (12.4)	53.4 (13.5)	
Sex, *n* (%) M F	5 (45.5) 6 (54.5)	6 (33.3) 12 (66.7)	0.514
Smoking habit, *n* (%)	5 (45.5)	7 (38.9)	0.728
Hormonal compensation, *n* (%)	8 (72.7)	17 (94.4)	0.100
Treatment with tapazole, *n* (%)	3 (27.3)	–	0.019
Horizontal deviation angle, mean (SD)	46.0 (23.7)	–	n.a.
Diplopia, *n* (%) Constant Inconstant	11 (100) -	–	n.a.
Vertical angle deviation in P.P., mean (SD)	–	24.3 (8.3)	n.a.
Diplopia, *n* (%) Constant Inconstant	–	18 (100) -	n.a.
Vertical angle deviation in D.P., mean (SD)	–	15.8 (6.4)	n.a.
Diplopia, *n* (%) Constant Inconstant	- -	14 (77.8) 4 (22.2)	n.a.
**7 days**			
CFA for near	16.2 (5.8)	20.1 (7.2)	0.118
CFA for distance	9.5 (4.7)	12.9 (6.5)	0.111
DFA for near	5.3 (2.4)	5.1 (2.7)	0.868
DFA for distance	3.5 (3.0)	5.3 (3.9)	0.158

M, male; F, female; P.P., primary position; D.P., down-gaze position; SD, standard deviation; CFA, convergence fusional amplitude; DFA, divergence fusional amplitude. n.a. = not applicable.

**Figure 1:**
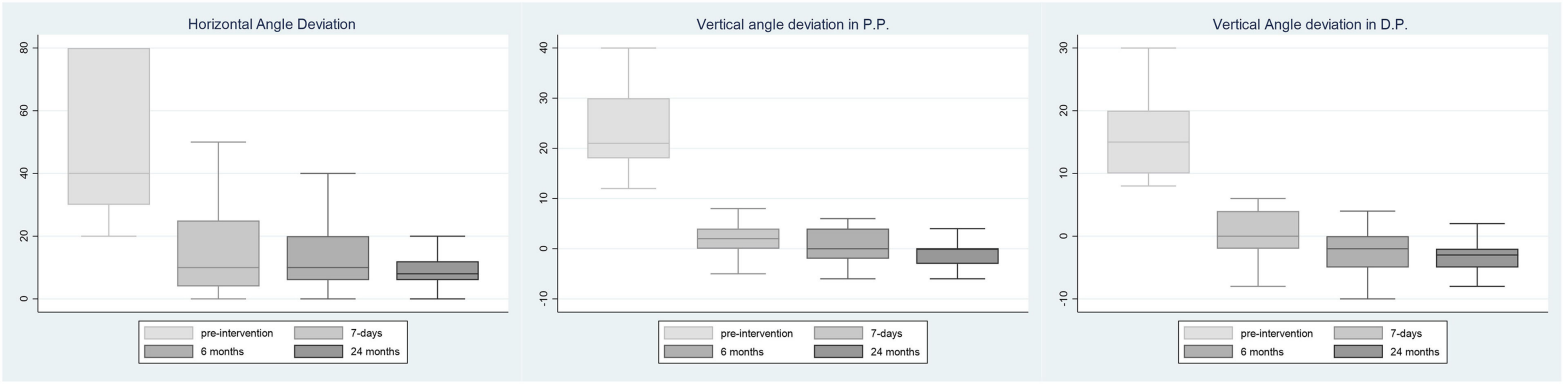
Deviation angle at four time points. Deviation angle modifications at 7 days, 6 months, and 24 months, with respect to pre-surgery in Group A and Group B, respectively. P.P., Primary Position; D.P., Down-Gaze Position.

**Figure 2:**
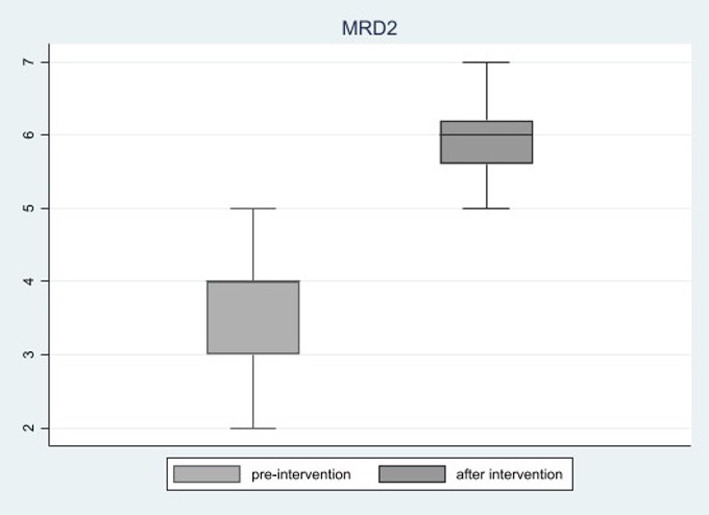
MRD2. MRD2 changes 6 months after surgery in Group B. MRD2: Margin Reflex Distance - 2.

Looking at deviation angle modifications across the four time points, as for Group A, the horizontal angle deviation in primary position (P.P.) significantly reduced from baseline (*p* < 0.001). A significant reduction occurred 7 days after surgery (46 ± 23.7 *vs*. 14.9 ± 14.3, *p* < 0.001), while the reduction then leveled off at 6 and 24 months, respectively. In Group B, instead, the vertical angle in P.P. also significantly decreased from baseline (*p* < 0.001). A significant reduction occurred both 7 days after surgery (24.3 ± 8.3 *vs*. 2.2 ± 3.2, *p* < 0.001) and between 7 days and 6 months (2.2 ± 3.2 *vs*. 0.6 ± 3.3, *p* = 0.040). As for the down-gaze position (D.P.), we observed a similar behavior. An inversion (right/left or left/right) of deviation angle in P.P. was observed in two patients at 7 days, in five patients at 6 months, and in seven patients at 24 months. In D.P., at 7 days, 5 patients showed an inversion of the vertical angle of deviation; at 6 months, 11 patients; and at 24 months, 12 patients. The whole data are reported in **Table 2** and **Figure 1**. Moreover, in this latter subgroup, MRD2 significantly increased 6 months after operation as compared to pre-surgery (mean 5.9 mm ± 0.8 *vs*. 3.5 ± 1.1, *p* < 0.001), as reported in **Figure 2**.

**Table 2:** Changes in angle deviation at the three time points after surgery in the two subgroups (*n* = 29).

Group A (*n* = 11)								
	**Baseline**	**7gg**	**6 months**	**24 months**	***p* ^overall^**	***p* ^(pre-7 gg)^**	***p* ^(7gg–6 m)^**	***p* ^(6m–24m)^**
Horizontal angle deviation in P.P., mean (SD)	46 (23.7)	14.9 (14.3)	13.6 (12.4)	8.4 (5.9)	<0.001	<0.001	0.381	0.055
**Group B (*n* = 18)**								
Vertical angle deviation in P.P., mean (SD)	24.3 (8.3)	2.2 (3.2)	0.6 (3.3)	−0.9 (3.2)	<0.001	<0.001	0.040	0.089
Vertical angle deviation in D.P., mean (SD)	15.8 (6.4)	0.3 (3.5)	−2.4 (3.7)	−3.4 (3.0)	<0.001	<0.001	0.002	0.453

P.P., primary position; D.P., down-gaze position; SD, standard deviation.

**Figure 3:**
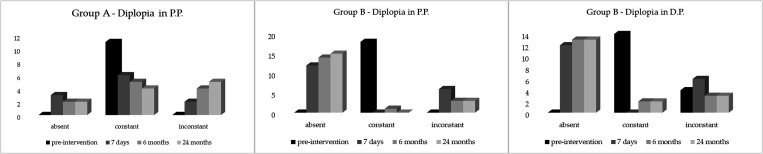
Trend of diplopia at four time points. Changes in diplopia from baseline (pre-surgery), up to 24 months’ follow-up in the two groups (Group A on the left and Group B in the middle and on the right). P.P.: Primary Position; D.P. Down-Gaze Position; MRD2.

At baseline, as aforementioned, all patients were characterized by diplopia, independent from the type of treatment. In Group A, in all cases, diplopia at baseline was constant, as well as in P.P. in Group B, while as for D.P., diplopia was inconstant in 4 of 18 cases. Diplopia cases reduced in both subgroups throughout the different time points, even though, as reported in **Tables 3** and **4**, no significant difference emerged at 6 and 24 months as compared to immediately post-surgery (**Figure 3**).

**Table 3:** Changes in diplopia at 6 and 24 months as compared to 7 days after surgery in the two subgroups (*n* = 29).

Group A (*n* = 11)	6 months				24 months			
**7 days**	Absent	Constant	Inconstant	*p*	Absent	Constant	Inconstant	*p*
Diplopia, *n* (%) Absent Constant Inconstant	2 0 0	0 5 0	1 1 2	0.368	2 0 0	- 4 0	1 2 2	0.223
**Group B (*n* = 18)**								
**P.P.**								
Diplopia, *n* (%) Absent Constant Inconstant	12 - 2	- - 1	- - 3	0.500	10 - 5	- - -	2 - 1	0.453
**D.P.**								
Diplopia, *n* (%) Absent Constant Inconstant	9 - 4	1 - 1	2 - 1	1.000	9 - 4	1 - 1	2 - 1	1.000

P.P., primary position; D.P., down-gaze position.

**Table 4:** Changes in diplopia treatable with prisms at 6 and 24 months as compared to 7 days after surgery in the two subgroups (*n* = 29).

Group A (*n* = 11)	6 months			24 months		
**7 days**	Yes	No	*p*	Yes	No	*p*
Diplopia treatable with prisms, *n* (%) Yes No	4 1	- 6	1.000	4 4	- 3	0.125
**Group B (*n* = 18)**						
**P.P.**						
Diplopia treatable with prisms, *n* (%) Yes No	4 -	2 12	0.500	1 1	5 11	0.219
**D.P.**						
Diplopia treatable with prisms, *n* (%) Yes No	1 3	4 10	1.000	1 4	4 9	1.000

P.P., primary position; D.P., down-gaze position.

In Group A, postoperative constant diplopia was observed in six patients, in two of whom, diplopia was treatable with prisms at 7 days; in the remaining four cases, the diplopia in three cases was treatable with prisms at 24 months. In Group B, constant diplopia did not occur at 7 days while six patients complained about inconstant diplopia in P.P. and in D.P., all of them corrigible with prisms. Two patients showed constant diplopia in D.P. at 6 and 24 months, corrigible with prisms. In P.P., one patient complained about constant diplopia, treatable with prisms at 6 months, which improved over time (at 24 months) (see **Tables 3**, **4**, **Figure 3**).

## Discussion

Approximately 0.6%–20% of patients with TAO will require strabismus surgery (6, 7). Although strabismus surgery aims to restore binocular single vision (BSV), an optimal outcome would be challenging to obtain (6, 7, 14).

Surgery is primarily focused on either establishing or improving BSVF in primary gaze and reading position. In such context, the evaluation of fusional vergences may be useful to predict the chance of postoperative compensation of potential hypo- or hypercorrection.

Prior to surgery, the angle of deviation should be stable for at least 4 to 6 months. Moreover, the thyroid function should be within the reference range and orbitopathy was inactive. In fact, operating during the active phase of TAO usually results in significant instability in surgical outcomes (15, 16).

Patients should be informed thoroughly to have realistic expectations. Likely more than one operation is needed. After strabismus surgery, BSV may be achieved only in primary and reading positions, proptosis may increase, and the lid lag might worsen (17). The frequency of reoperation in adults with TAO strabismus is relatively high, up to 26% (18, 19).

Undercorrection is the most common reported complication of horizontal strabismus correction, but simultaneous conjunctiva and Tenon’s recession seems to improve the outcome (11, 12, 20). Late overcorrection may occur instead after inferior rectus recession. A postoperative mean drift toward overcorrection (from 1.9^Δ^ to 3^Δ^) has been described (8, 13). Several elements have been reported as significant prognostic factors for postoperative overcorrection, including duration and severity of orbitopathy, impaired contralateral elevation, and underestimation of increased ipsilateral superior rectus tone (21). Moreover, the progression of underlying thyroid myopathy after strabismus surgery, even in cases with a stable angle of deviation for 6 months, may result in late postoperative instability of deviation. In addition, anatomical causes, as well as an inadequate muscle scleral fixation, may lead to either muscle slippage or posterior shifting of inferior rectus scleral insertion. Predisposing factors for these postoperative complications are the gravitational forces, the short arc of contact of inferior rectus muscle, and the thickened tenon beneath the inferior rectus muscle in TAO patients (22).

Down-gaze diplopia due to an early or late inferior rectus under-action, with vertical strabismus in down-gaze after inferior rectus weakening, is usually poorly tolerated. The reversal of angle deviation has been often related to an overlooked preoperative incomitance between primary position and down-gaze, often the result of the mechanical imbalance between opposing muscle groups (23). Moreover, inferior rectus muscle recession tends to retract the lower eyelid due to the anatomical connections with Lockwood ligament and lower eyelid retractors. Several surgical techniques have been recommended to correct lower lid retraction (24).

In Group A, an immediate significant improvement of the horizontal angle deviation was observed from baseline at 7 days from surgery without modification over time. A further but not significant decrease was, however, observed at 6 and 24 months. The result, in agreement with the data reported by some authors, and despite the simultaneous conjunctiva and Tenon’s recession, is an undercorrection relatively stable over time (11, 12, 20). In Group B, a significant reduction of the angle of deviation in P.P. and in D.P. was observed 7 days after surgery, with a further significant reduction at 6 months with an overcorrection and an inversion right/left or left/right of the angle of deviation in some cases mainly in D.P. (**Figure 1**). The angle leveled off 6 months after surgery without significant further modification at 24 months in P.P. and D.P. The tendency toward overcorrection, as already reported after inferior rectus recession, seems limited to the first 6 months post-surgery with subsequent stabilization of the angle of deviation.

Despite the infratarsal lower eyelid retractor lysis, performed simultaneously with inferior rectus muscle recession, a significant increase in MRD2 was observed at 6 months after surgery with a significant lower eyelid ptosis and scleral show. The preoperative values are nevertheless below the normal range (4–5 mm) as a result of a forced down-gaze eye position (25).

As expected, a significant post-surgical treatment improvement of diplopia was observed in both groups and, in Group B, also in D.P. Often the diplopia after surgery was absent or inconstant and treatable with prisms without significant modification over time except for four cases in Group A that complained of constant diplopia at 24 months; of these, three were treatable with prisms, and two cases in Group B that showed constant diplopia were treatable with prisms.

The patients with constant diplopia were later reoperated on and the reoperation rate was of 4 out 11 (36.6%) cases in Group A and 2 out 18 (11.1%) cases in Group B (constant diplopia in D.P.) according to other reports (18, 19).

In conclusion, when we look at the horizontal and vertical surgery of TAO strabismus separately, the result is an undercorrection relatively stable over time for horizontal surgery and a good immediate response with an overcorrection, within and not later than 6 months, for vertical strabismus. The horizontal surgery has a higher reoperation rate and the lower eyelid ptosis is a significant functional and aesthetic complication of inferior rectus weakening. This study has some limitations, mainly the retrospective nature of the study and its relatively small sample size. Further prospective and hopefully multicentric studies are needed to validate our results.

## Data availability statement

The datasets presented in this study can be found in online repositories. The names of the repository/repositories and accession number(s) can be found below: https://github.com/piaclarapafundi/TAO-STRABISMUS.git.

## Ethics statement

The studies involving human participants were reviewed and approved by the Head and Neck Institutional Review Board (approval ID; 18/2020). The patients/participants provided their written informed consent to participate in this study.

## Author contributions

GS conceived and designed the work, played an important role in interpreting the results, revised the manuscript, and approved the final version. RM acquired data, drafted the manuscript, and approved the final version. AS acquired data, drafted the manuscript, and approved the final version. CF acquired data, played an important role in interpreting the results, revised the manuscript, and approved the final version. PP designed the work, revised the manuscript, and approved the final version. All authors contributed to the article and approved the submitted version.

## Acknowledgments

The authors want to thank “Ministero della Salute- Ricerca Corrente 2022” and Associazione Oncologia Oculare Onlus for funding support.

## Conflict of interest

The authors declare that the research was conducted in the absence of any commercial or financial relationships that could be construed as a potential conflict of interest.

## Publisher’s note

All claims expressed in this article are solely those of the authors and do not necessarily represent those of their affiliated organizations, or those of the publisher, the editors and the reviewers. Any product that may be evaluated in this article, or claim that may be made by its manufacturer, is not guaranteed or endorsed by the publisher.
